# Evaluation of a multi-component, non-pharmacological intervention to prevent and reduce sleep disturbances in people with dementia living in nursing homes (MoNoPol-sleep): study protocol for a cluster-randomized exploratory trial

**DOI:** 10.1186/s12877-020-01997-8

**Published:** 2021-01-12

**Authors:** Martin N. Dichter, Almuth Berg, Jonas Hylla, Daniela Eggers, Denise Wilfling, Ralph Möhler, Burkhard Haastert, Gabriele Meyer, Margareta Halek, Sascha Köpke

**Affiliations:** 1 Institute of Nursing Science, University Hospital of Cologne, Gleuler Straße 176-178, D-50935 Cologne, Germany; 2 Neurodegenerative Diseases (DZNE), Witten, Stockumer Straße 12, 58453 Witten, Germany; 3 School of Nursing Science, Witten/Herdecke University, Stockumer Straße 12, 58453 Witten, Germany; 4 Institute for Health and Nursing Science, Medical Faculty, Martin Luther University Halle-Wittenberg, 06112 Halle (Saale), Germany; 5 Institute of Social Medicine and Epidemiology, University of Lübeck, Ratzeburger Allee 160, 23562 Lübeck, Germany; 6 Institute for Health Services Research and Health Economics, Center for Health and Society, Faculty of Medicine, Heinrich-Heine University Düsseldorf, Moorenstraße 5, 40225 Düsseldorf, Germany; 7 School of Public health, Bielefeld University, Universitätsstraße 25, 33615 Bielefeld, Germany; 8 mediStatistica, Neuenrade, Germany

**Keywords:** Dementia, Sleep disturbances, Complex intervention, Cluster-randomized controlled trial, Nursing home, Nursing

## Abstract

**Background:**

Sleep problems are highly prevalent in people with dementia. Nevertheless, there is no “gold standard” intervention to prevent or reduce sleep problems in people with dementia. Existing interventions are characterized by a pronounced heterogeneity as well as insufficient knowledge about the possibilities and challenges of implementation. The aim of this study is to pilot and evaluate the effectiveness of a newly developed complex intervention to prevent and reduce sleep problems in people with dementia living in nursing homes.

**Methods:**

This study is a parallel group cluster-randomized controlled trial. The intervention consists of six components: (1) the assessment of established sleep-promoting interventions and an appropriate environment in the participating nursing homes, (2) the implementation of two “sleep nurses” as change agents per nursing home, (3) a basic education course for nursing staff: “Sleep problems in dementia”, (4) an advanced education course for nursing staff: “Tailored problem-solving” (two workshops), (5) workshops: “Development of an institutional sleep-promoting concept” (two workshops with nursing management and sleep nurses) and (6) written information and education material (e.g. brochure and “One Minute Wonder” poster). The intervention will be performed over a period of 16 weeks and compared with usual care in the control group.

Overall, 24 nursing homes in North, East and West Germany will be included and randomized in a 1:1 ratio. The primary outcome is the prevalence of sleep problems in people with dementia living in nursing homes. Secondary outcomes are quality of life, quality of sleep, daytime sleepiness and agitated behavior of people with dementia, as well as safety parameters like psychotropic medication, falls and physical restraints. The outcomes will be assessed using a mix of instruments based on self- and proxy-rating. A cost analysis and a process evaluation will be performed in conjunction with the study.

**Conclusions:**

It is expected that the intervention will reduce the prevalence of sleep problems in people with dementia, thus not only improving the quality of life for people with dementia, but also relieving the burden on nursing staff caused by sleep problems.

**Trial registration:**

Current controlled trials: ISRCTN36015309. Date of registration: 06/11/2020.

## Background

Due to demographic change, the population of older people will continue to increase. It is expected that by 2050 the number will double from 1 to 1.5 million to around 3 million [1]. With increasing age, the prevalence of dementia increases. In Germany, prevalence rates for people aged ≥65 `s have been estimated at 8.8% and the annual incidence at 1.9% [2, 3]. Dementia is a clinical syndrome characterized by cognitive, neuropsychiatric and functional symptoms. It involves difficulties in memory, disturbances in language, psychological and psychiatric changes as well as impairments in activities of daily living [4].

People with dementia have increased sleep problems, which could be defined as a decreased total sleep time of one quarter of the individual total nocturnal sleep appertaining to their premorbid nocturnal sleep pattern or, if this is not known, a sleep pattern of less than six hours between 9:00 pm and 6:00 am [5]. The main reasons for sleep problems are advancing age, chronic illnesses, mobility restrictions, reduced brain performance and taking medication [6–9].

A German study showed a 23%prevalence of sleep disturbances for people with dementia living in nursing homes [10], and a recent meta-analysis demonstrated a 20% prevalence in validated proxy-based questionnaires and a prevalence of 38% for any symptom of sleep disturbances [11].

Evidence demonstrates that poor sleep quality is associated with harmful consequences regarding physical health, mental health disorders and quality of life [12, 13]. Sleep problems in people with dementia are often accompanied by agitation or aggressive behavior [14] and are associated with significant caregiver distress [15]. In nursing home care, the relationship between nurses and people with dementia results in a reciprocal interaction. The behavior and emotions of people with dementia affect nurses’ well-being and behavior, and vice versa [16]. This means that residents’ challenging behaviors lead to a lower quality of general health, reduced workability, high burnout levels [17], depression, anxiety and sleep problems for the nursing home staff [18]. As a consequence, this can result in higher staff sick leave and turnover, and increased costs [19].

To counteract challenging behavior, nursing home residents are often prescribed psychoactive drugs, including hypnotics. A study from the USA found that 47% of people with dementia in nursing homes received psychotropic medication [20]. The use of benzodiazepines and non-benzodiazepines is associated with an increased rate of falls and the associated increased risk of fractures [21, 22]. A Cochrane review showed that there are currently no effective pharmacological interventions to treat sleep problems in people with dementia in various settings [23]. Although regularly prescribed, drug therapies with, for example, antipsychotics, benzodiazepines or z-drugs are of unclear effectiveness and potential harm and therefore not considered to be the first choice for sleep disturbances in people with dementia in nursing homes [23].

In a Cochrane review currently being prepared on non-pharmacological interventions [24], multi-component or “complex” interventions, with contain multiple (interacting) components, are shown to have the strongest potential of being effective for improving sleep. The main challenge of interventions aiming to prevent or reduce sleep disturbances are the number of potential triggers or causes of sleep disturbances, the number and difficulty of behaviors and skills required by those delivering the intervention, the number of groups of organizational levels targeted by the intervention, the number and heterogeneity of outcomes, and the degree of flexibility or tailoring of the intervention [25, 26]. Based on these, complex interventions appear to be most appropriate for the multifactorial origin of sleep disturbances and the requirements for a tailored intervention.

In a recent systematic review [27], we described and summarized non-pharmacological complex interventions targeting sleep problems in nursing home residents. Frequently used components of complex interventions aiming to reduce sleep problems were: activating nursing home residents during daytime, creating bedtime routines, creating sleep-promoting night care, avoiding negative symptoms such as pain, itching, anxiety, and/or creating a sleep-promoting environment (e.g. concerning light, noise, temperature). Identified complex interventions showed a wide variance regarding the addressed influencing factors for sleep problems, the intervention components applied, and the intensity and duration of delivery. Therefore, we were not able to recommend one specific component of an intervention, but a whole bundle. However, the review recommends the rigorous development of non-pharmacological interventions to improve residents’ sleep in nursing homes, taking the potential barriers for implementation into consideration.

## Objectives

The aim of this trial is the piloting and evaluation of the effects of a newly developed complex intervention program to prevent and reduce sleep disturbances in people with dementia in nursing homes. The intervention was developed according to the UK Medical Research Council (MRC) framework for complex interventions [25] and is based on the results from an own systematic review [27], a theory of change [28] and a survey [10, 15] as well as qualitative interviews, observations and additional literature. This new complex intervention will be evaluated in an exploratory mixed-methods cluster-randomized controlled trial. Implementation fidelity as well as the promoting and inhibiting factors of the intervention’s implementation will be examined in an accompanying process evaluation [29, 30]. In addition, an evaluation of the implementation costs will be carried out.

## Study design

**Fig. 1:**
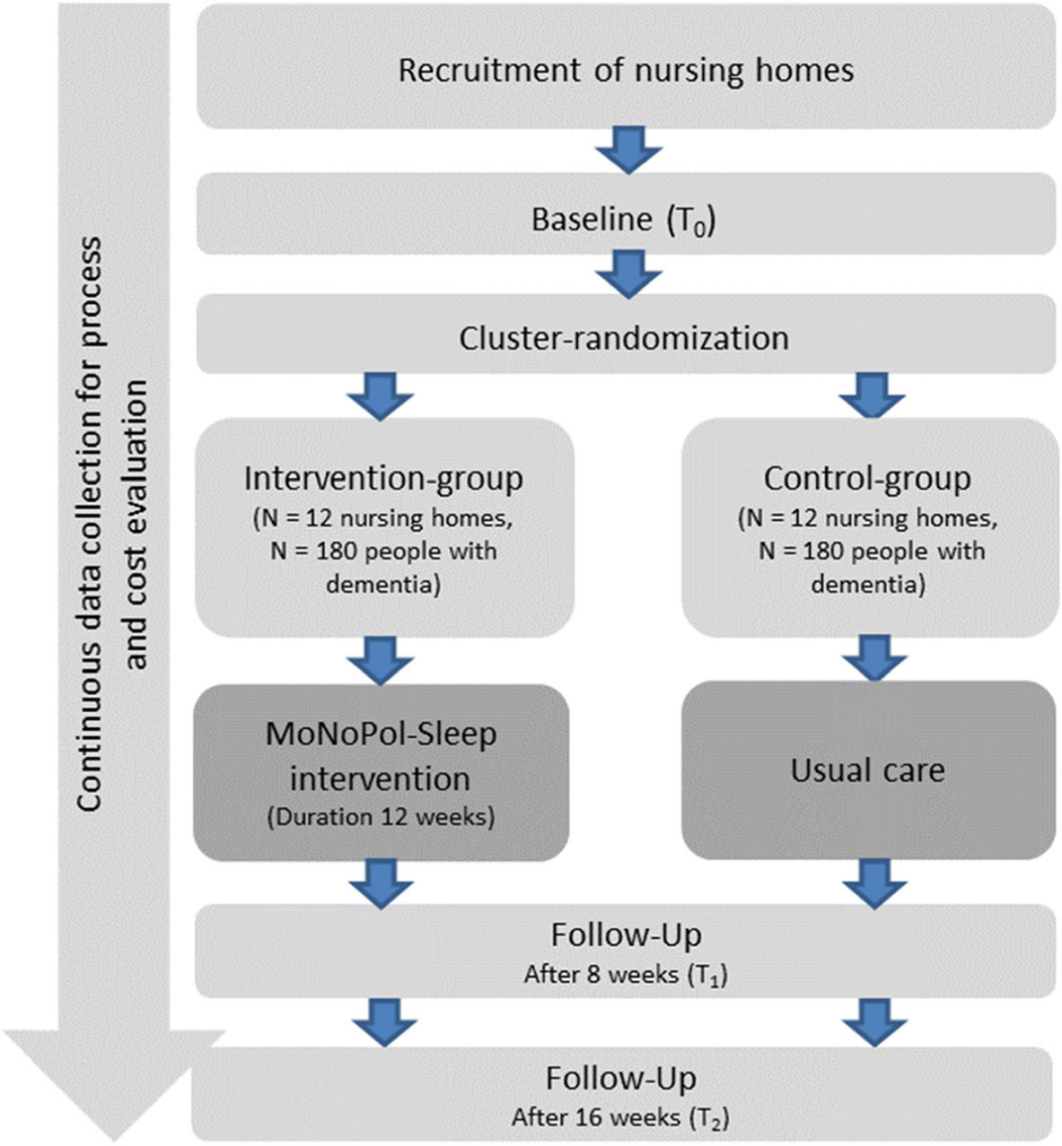
Flowchart for the cluster-randomized controlled trial

The MoNoPol-Sleep (multi-modal, non-pharmacological intervention for sleep disturbances in people with dementia living in nursing homes) trial is a cluster-randomized controlled trial with two parallel groups and a 16-week follow-up. A total of 24 nursing homes will be randomized either to (1) an intervention group receiving the complex intervention program or to (2) a control group receiving usual care (see Fig. 1). The expected start of recruitment for the study participants is March 2021.

## Methods: participants, interventions and outcomes

### Eligibility criteria

#### Cluster level

Nursing homes must have at least 50 residents to be included. Moreover, nursing homes must indicate that they have sufficient resources (staff and time) to conduct the study and to implement the intervention, and that no parallel dementia-specific care-related project is being conducted there during the MoNoPol-Sleep trial.

#### Resident level

The inclusion criteria for people with dementia living in nursing homes are a documented dementia diagnosis or a score of ≥3 on the Dementia Screening Scale (DSS) [31]. Further criteria are the presence of at least two sleep problems according to the Sleep Disorder Inventory (SDI) [32] and a length of stay ≥2 weeks in the respective cluster. Exclusion criteria are documented sleep apnea, REM-sleep-behavior disorders or respite care.

#### Nurse level

The following inclusion criteria for nurses will be applied: a contract for at least part-time (half-a-day) work and at least three night shifts during the last 3 months prior to data collection.

### Recruitment of clusters and study participants

Nursing homes will be recruited in the catchment regions of Lübeck (Northern Germany), Halle an der Saale (Eastern Germany) and Witten (Western Germany). Eight clusters per region will be included in the convenience sample. For the recruitment, each study center (Lübeck, Halle an der Saale, Witten) will use existing contacts to nursing homes in their area. In addition, nursing homes will be recruited by means of an information folder, announcements in relevant journals for long-term care in Germany, and the study website (www.monopol-sleep.de).

Nursing homes interested in participating in the study will be contacted by the study center via telephone or E-Mail and verbally informed about the study in a telephone call or during a personal visit. The aims and contents of the MoNoPol-Sleep study will be presented to the respective nursing home manager, who will decide whether to participate.

#### Who will take informed consent?

One staff member in each nursing home will be deployed as the project coordinator. Interested and eligible people with dementia and/or their legal representative will receive written study information provided by the project coordinators. Informed consent will be obtained by the project coordinators and sent to the respective study center.

### Intervention

The aim of the intervention is to prevent and reduce sleep problems of people with dementia by implementing person-centered care including a sleep-promoting care environment for each cluster.

#### Intervention description

##### Intervention group

**Fig. 2:**
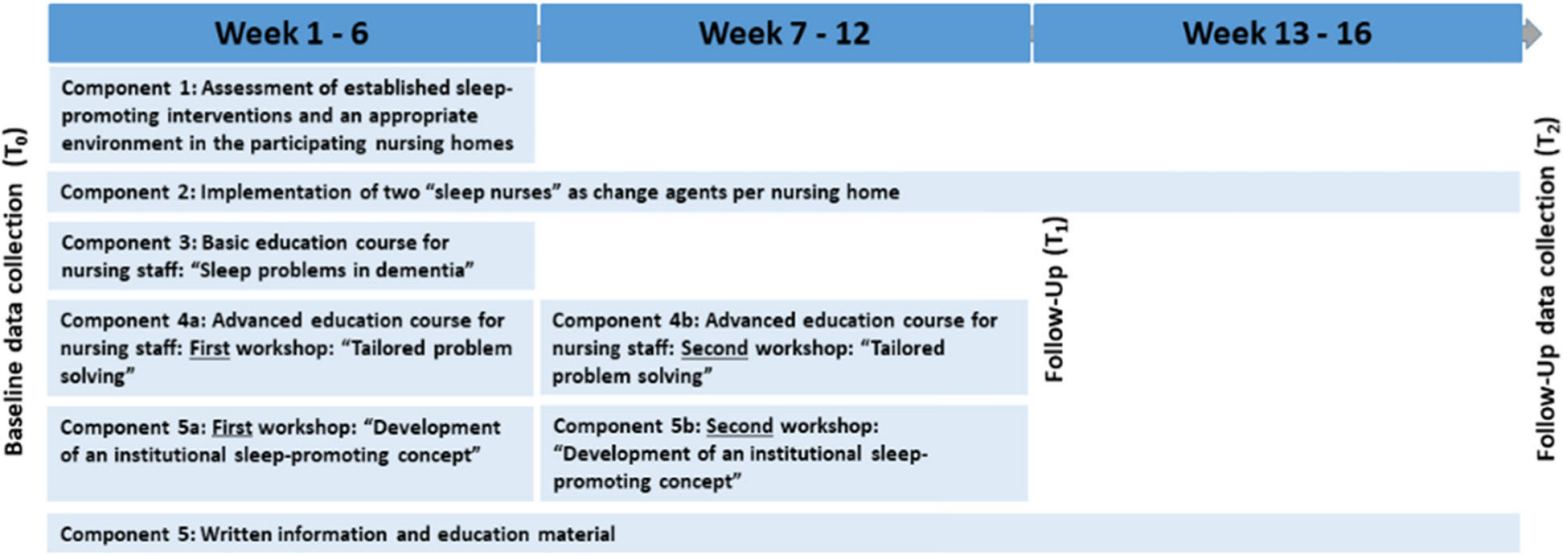
Schedule of the MoNoPol-Sleep intervention components

The intervention consists of six components, which will be implemented over a period of 16 weeks with the support of the research team (Fig. 2). The six components are described in detail in Table 1.

**Table 1:** Detailed description of the components of the MoNoPol-Sleep intervention to reduce and prevent sleep problems for people with dementia in nursing homes

No.	What? Procedures	Why? Rationale and theory	What? Materials	How? Modes of delivery	Who? Intervention provider	When and how much? Number of sessions etc.
1.	**Assessment of established sleep-promoting interventions and an appropriate environment in the participating nursing homes** ▪ This assessment is based on interviews with the nursing staff (from night shifts) and a proxy assessment (via structured observations) of implemented interventions to promote the sleep of people with dementia.	▪ Nursing homes are heterogeneous in terms of the existing prevalence of sleep problems and implemented interventions to avoid or reduce sleep problems [10, 33]. ▪ To ensure that the MoNoPol-Sleep intervention can be individually tailored to the needs of the participating nursing home, an assessment will be carried out.	▪ Self-developed structured observation checklist and interview guide (which was already tested and adapted during the intervention development) ▪ Structured feedback as part of intervention component No. 3	▪ Observation on site (participating nursing home) and face to face interviews	▪ Research staff	▪ One proxy assessment (on site observation between 5 p.m. and 2 a.m.) and interviews with a minimum of four nurses (two day shift and two night shift) ▪ First week of the intervention phase
2.	**Implementation of two “sleep nurses” as change agents per nursing home.** ▪ During the intervention phase, the sleep nurses (one nurse from the day shift and one nurse from the night shift) act as contact persons, multipliers, coordinators, change agents and as implementers of person-centered sleep promotion. ▪ For this purpose, the sleep nurses receive in-depth knowledge about sleep promotion, implementation strategies with regard to the MoNoPol-Sleep interventions and problem-solving strategies. ▪ In addition, the sleep nurses can reflect on their actions in coaching sessions and develop new problem-solving strategies.	▪ Local praxis development champions are a crucial factor to implement person-centered care interventions [34, 35] ▪ Sleep nurses are important as contact persons, change agents and local champions. Such champions are recommended for the implementation of complex interventions [36].	▪ Written education material ▪ Written profile of the sleep nurses	▪ Education sessions (both sleep nurses together for each nursing home) ▪ Coaching sessions (both sleep nurses together for each nursing home)	▪ Research staff	▪ Education: approx. Two hours of education as part of component No. 3 ▪ Coaching: approx. One hour after each workshop of the components No. 4 and 5
3.	**Basic education course for nursing staff: “Sleep problems in dementia”.** ▪ The education session is divided into three parts: ▪ Part 1 aims to provide information on the following topics: information on the background, course, schedule and responsibilities of the MoNoPol-Sleep trial, principles of person-centered care, sleep and sleep problems of people with dementia. Moreover, evidence-based information regarding six strategies to promote sleep of people with dementia will be presented. These strategies are: (1) Activation of people with dementia during the day, (2) Review and, if necessary, adjustment of the “going to bed routine”, (3) Review and, if necessary, adjustment of the night care (e.g. change of position routine), (4) Freedom from symptoms (e.g. with regard to thirst, pain, itching, anxiety), (5) Reflection of the appropriateness of sleep medication (6) Review and, if necessary, adjustment of the sleeping environment (e.g. light, sounds, temperature) ▪ The results of the assessment of established sleep-promoting interventions (component No. 1) will be presented in Part 2. ▪ Part 3 aims to provide information on the function and tasks of the new role of a sleep nurse (component No. 2) as change agent in each nursing home (intervention group).	▪ Information regarding six strategies to promote sleep of people with dementia will be based on three reviews [23, 27, 37]. ▪ To give the nursing staff basic information about sleep problems in dementia as well as the schedule of the upcoming interventions. ▪ Sleep nurses need further information to fulfill their new role.	▪ Written education material ▪ Information brochure (component No. 6)	▪ Group session	▪ Research staff	▪ One session divided into three parts: ▪ Part 1 takes approx. 45 min, while Part 2 takes approx. 45 min and Part 3 lasts approx. Two hours ▪ Second week of intervention
4.	**Advanced education course for nursing staff: “Tailored problem solving” (two workshops).** ▪ Two workshops which aim to support the implementation of person-centered sleep promotion and problem-solving competencies. ▪ In both workshops, one case of a resident with sleep problems from the facility will be discussed and solutions for improved sleep promotion sought. ▪ Each workshop contains the following seven steps: (1) Introduction, (2) Presentation of one resident as a case with sleep problems by the sleep nurse, (3) Prioritizing of care problems by all workshop participants, (4) Information about potential reasons for sleep problems by the moderating researcher, (5) Analysis of potential influencing factors for case-specific sleep problems by all workshop participants, (6) Formation of hypotheses for understanding, (7) Development and consensus on an action plan.	▪ Based on a theory of change which was developed during the MoNoPol-Sleep intervention development: Sleep problems and their causes are individual for each person with dementia. ▪ Nursing home staff needs support in problem solving which take the available resources of people with dementia and the care environment into account. ▪ The seven steps of the workshop are based on the available knowledge for conducting case conferences [38–40].	▪ Direction plan for the workshop ▪ Digital presentation ▪ Written education material ▪ Self-developed, standardized case analysis chart ▪ Moderator manual including e.g. list of causes for sleep problems, list of symptoms of sleep problems)	▪ Group sessions	▪ Research staff ▪ Sleep nurses	▪ Both workshops take approx. 1.5 h each ▪ The first workshop will be held after component No. 3 and by the 6th week of intervention at the latest ▪ The second workshop will be held between the 7th and 12th week of intervention
5.	**Workshops: “Development of an institutional sleep-promoting concept” (two workshops with nursing management and sleep nurses).** ▪ Two workshops aimed at the development and implementation of an organizational, specific action plan to promote sleep. ▪ The first workshop contains the following four steps: (1) Introduction, (2) Knowledge refreshment regarding the results of the assessment of established sleep-promoting interventions (component No. 1) and six strategies to promote sleep of people with dementia (see component No. 3), (3) Identification of organizational structures and processes which promote or hinder the sleep promotion, (4) Development and consensus on an action plan. ▪ The second workshop contains three steps: (1) Introduction, (2) Identification of organizational structures and processes which promote or hinder the implementation of the action plan (workshop 1), (3) Development and consensus on the implementation of the action plan.	▪ Based on a theory of change which was developed during the MoNoPol-Sleep intervention development: Nursing homes are heterogeneous in terms of opportunities to provide person-centered sleep promotion.	▪ Self-developed, standardized workshop protocol including resources for the moderator (e.g. action plan form)	▪ Group sessions	▪ Research staff ▪ Sleep nurses	▪ Both workshops take approx. 2.5 h each ▪ The first workshop will be held after component No. 3 and by the 6th week of intervention at the latest ▪ The second workshop will be held between the 7th and 12th week of intervention
6.	**Written information and education material (e.g. brochure and “One Minute Wonder” poster).** ▪ A series of eight “One Minute Wonder” posters addressing restful sleep, the identification of sleep problems and six strategies to promote sleep of people with dementia (see component No. 3). ▪ Two individual nursing home “One Minute Wonder” posters which will contain the nursing home’s specific action plan and its implementation (component No. 5). ▪ Information brochure which contains evidence-based information on sleep and sleep problems of people with dementia, six strategies to promote sleep of people with dementia (see component No. 3) and education material.	▪ See components No. 1 to 5	▪ “One Minute Wonder” poster ▪ Information brochure	▪ Not applicable	▪ Research staff	▪ Each ward of the participating nursing homes will receive the poster in weekly rhythm ▪ The first posters of the series are distributed on the wards after the basic education course (component No. 3) ▪ After completion of the basic education course, the staff of the nursing home will receive a brochure

#### Explanation for the choice of comparators

As the current study aims to explore the feasibility and effectiveness of a novel intervention, we have chosen usual care as the comparator. In a further, fully powered trial aiming to assess the efficacy of the MoNoPol-Sleep intervention, optimized usual care might be chosen as comparator.

#### Criteria for discontinuing or modifying allocated interventions

Not applicable for this non-pharmacological intervention, but nursing homes can leave the study at any time and may withdraw consent.

#### Strategies to improve adherence to interventions

To ensure involvement and adherence of participating nursing homes the research team will provide interested nursing homes with comprehensive information about the study that is specific for the target group. The short intervention period of 16 weeks allows the nursing homes to be closely monitored during data collection and intervention delivery. After completion of the follow-up survey, the control group will receive one to two hours’ training on complex interventions for sleep promotion in people with dementia and will also be provided with corresponding information material developed for the intervention group.

#### Relevant concomitant care permitted or prohibited during the trial

The MoNoPol-Sleep intervention does not replace any part of health care. In order to promote the sleep of people with dementia, the intervention raises awareness, knowledge and skills of nursing home staff with regard to sleep promotion. Furthermore, the current sleep-relevant practices and processes within the nursing home will be reflected.

#### Provisions for post-trial care

Not applicable (see adverse event reporting and harms).

### Outcomes

To investigate the intervention effects, quantitative measurements will be used to measure variables regarding people with dementia and nursing staff. Table 2 summarizes all the outcome instruments.

**Table 2:** Outcome instruments

People with dementia				
Variable	Instrument	Source	Measurement points	Type of variable
Demographic variables (e.g. documented sleep apnea, documented dementia diagnosis, Dementia Screening Scale, length of stay in nursing home)	Single items	Nursing records	T0	Inclusion and exclusion criteria
Demographic Variables (e.g. age, sex, care dependency level^a^)	Single items	Nursing records	T0	Control variables
Sleep Disorders	Sleep Disorder Inventory [32]	Retrospective rating by registered nurse (night shift)^c^	T0, T1, T2	Primary outcome, Inclusion criterion
Objective Sleep Measures^b^	Algorithm to analyze actigraphic data with respect to sleep patterns [41, 42]	Actigraphy	T0, T2	Secondary outcome
Quality of Sleep	Pittsburgh Sleep Quality Index [43]	Self-rating by participating people with dementia^d^	T0, T2	Secondary outcome
Daytime Sleepiness	Essener Questionaire of Age and Sleepiness in the Elderly (EFAS) [44]	Retrospective rating by registered nurse (day shift)	T0, T2	Secondary outcome
Quality of Life	QUALIDEM 2.0 [45, 46]	Retrospective rating by registered nurse (night shift)^c^	T0, T2	Secondary outcome
Agitation	Cohen-Mansfield Agitation Inventory [47]	Retrospective rating by registered nurse (day shift)^b^	T0, T2	Secondary outcome
Accidental falls	Single item	Nursing documentation	T1, T2	Control variable
Physical restraint use	Single item	Nursing documentation	T1, T2	Control variable
Psychotropic medication	Single items	Nursing documentation	T1, T2	Control variable
**Staff**				
**Variable**	**Instrument**	**Source**	**Measurement** **points**	**Type of variable**
Demographic variables (e.g. age, sex)	Single items	Staff questionnaire	T0	Control variable
Nurses sleep-related distress	Sleep Disorder Inventory [32]	Retrospective rating by registered nurse (night shift)	T0, T1, T2	Secondary outcome
Nurses distress caused by residents’ challenging behavior	Residents’ Challenging Behavior–related Distress Index [17]	Retrospective rating by registered nurse (day & night shift)	T0, T2	Secondary outcome
		**Cost analysis**		
**Variable**	**Instrument**	**Source**	**Measurement points**	**Type of variable**
Costs related to the implementation of the intervention and the predefined outcomes	Self-developed cost protocol [48]	Documentation by trained external study assistants	Ongoing	Cost outcome

^a^Care levels based on the assessment of the experts of the medical service of the health insurance funds

^b^Only subgroup of eight nursing homes of the Lübeck Study Centre

^c^Retrospective assessment (of the last 2 weeks before data collection) by nursing professionals. Here, the nursing professionals are instructed by a member of the scientific team

^d^Self-assessment = people with dementia guided by a member of the research team

T0 = baseline before randomization, T1 = follow-up after 8 weeks, T2 = follow-up after 16 weeks

For the process evaluation, quantitative data from nursing home managers, staff and the process documentation (intervention delivery and implementation) as well as qualitative staff interviews (individual and focus group interviews) will be assessed (Table 3).

**Table 3:** Process evaluation: data collection methods

Parameter	Material	Measurement points	Elements of process evaluation
Recruitment description	▪ Documentation of the recruitment process ▪ Survey of reasons for participation (nursing home management/nursing service management) ▪ Survey of reasons for non-participation/withdrawal (nursing home management/nursing service management)	T0	Recruitment
Description of the context factors of the nursing homes	▪ Existence of concepts or standards for dealing with sleep or promoting sleep (structured survey, nursing service management)	T0, T2 (only control group)	Description of the cluster (Context)
	▪ Culture in the nursing homes (Organizational Readiness for Implementing Change (ORIC)) [49]. ▪ Person-centered care (Person-centered climate questionnaire (staff version) (PCQ-S)) [50]. ▪ Team effort (Assessment of Interprofessional Team Collaboration Scale (AITCS)) [51]. ▪ Sample: nursing services and residential area managers, as well as 20% of all nursing staff per cluster	T0, T2	
Implementation of the intervention components	▪ Information about the needs assessment for each cluster (e.g. number of stakeholders involved, content) ▪ Number, frequency and content of the different components delivered to each cluster (including duration, number and function of participants, topics, necessary adjustments (type, reasons), deviations from the protocol), i.e.: ▪ Educational courses (basic and advanced) offered per cluster ▪ Workshops offered per cluster ▪ Training and support delivered to the change agents ▪ Other components offered	Implementation phase, T1, T2 (only intervention group)	Implementation of the intervention (Delivery)
Adoption of the intervention components in the nursing homes	▪ Content of the sleep-related care concept (document analysis)	T1, T2	Implementation of the intervention (Response)
	▪ Number (rate) of participants attending the workshops per cluster (documentation)	Implementation phase	
	▪ Number of workshops and internal training planned and carried out in the context of the intervention	T1, T2	
	▪ Changes in procedures and processes as a result of the interventions’ implementation (e.g. assessments)	T1, T2	
Change processes in the nursing homes	▪ Changes in procedures and processes due to the implementation of the sleep concept in each cluster (planned vs. implemented)	T1, T2 (only intervention group	
	▪ Changes regarding sleep promotion in the care plans of people with dementia (document analysis, *n* = 5 residents with sleep problems per cluster)	T2 (only intervention group)	
	▪ Performance of care plans for sleep promotion (participating observations, 2 residents with sleep problems from selected nurses (2 per cluster))	T2 (only intervention group)	
	▪ Perception of changes in sleep promotion from people with dementia or their representatives, relatives or legal guardians (interviews, 4 per cluster)	T2 (only intervention group)	
Inhibiting / promoting factors and contextual conditions	▪ Perspective of the target groups (managers, nurses actively involved in the intervention; interviews)	T2 (only intervention group)	

T0 = baseline before randomization, T1 = follow-up after 8 weeks, T2 = follow-up after 16 weeks

Data collection will take place before randomization and intervention implementation (T0, baseline), then again after 8 weeks of intervention (T1, Follow-Up) and after completion of the intervention at 16 weeks (T2, Follow-Up). Process and cost data will be collected ongoing throughout the trial and after completion of the intervention (Fig. 1).

#### Primary outcome

The primary endpoint of the MoNoPol-Sleep trial is the prevalence of at least two sleep problems in people with dementia within the last 2 weeks, assessed with the Sleep Disorder Inventory (SDI) [32]. This instrument (SDI) is based on the Neuropsychiatric Inventory Nursing Home (NPI-NH) [32] and describes the frequency and severity of sleep problems by querying the following sleep problems (German version): (1) “Difficulty falling asleep”, (2) “Getting up in the night”, (3) “Walking around, walking up and down or engaging in inappropriate activities at night”, (4) “Waking up at night, getting dressed with the intention of going outside, thinking that it is morning and it is time to start the day”, (5) “waking up too early in the morning”, (6) “sleeping excessively during the day”, and (7) “other nocturnal behaviors that disturb you”. The presence of these sleep problems is assessed as “yes” or “no” respectively. This allows the detection of existing sleep problems (primary endpoint). Possible missing values will be interpreted as “No”.

Furthermore, the sleep problems are assessed in terms of their frequency and severity. A total value between 0 and 84 is formed by multiplication of frequency and severity data. Higher values correspond to more severe sleep problems (secondary endpoint). The proxy-assessment of sleep problems using Sleep Disorder Inventory (SDI) [32] is carried out exclusively by nurses who have done at least three night shifts in the last 14 days. These nurses will assess their own burden caused by the sleep problems of people with dementia, based on a five-level response scale from “not at all” to “very severe” of the SDI. The burden of the nurses measured in this way is a secondary endpoint in the present study.

In the course of the MoNoPol-Sleep intervention development, the SDI was translated into the German language according to Beaton’s recommendations [52]. This guideline for instrument translation consists of five comprehensive steps: (1) forward translation, (2) synthesis, (3) backward translation, (4) expert committee review, (5) pretesting the instrument. Within the first step (1) two translators with German as their native language and excellent English skills (one with a professional background in dementia care) translated the original version of the SDI. After the independent translation of the whole instrument into German, the translators created one consensus version (2). In the third step (3) two translators with English as their native language and excellent German language skills translated this version back into English independent from each other and reached a consensus again. They were blinded to the original version of SDI. Subsequently, an expert committee (4) consisting of all four translators, a registered nurse experienced in the care for older people during the night, an expert in the field of dementia research and the moderator (JH), who is a registered nurse and researcher, determined the final German SDI version. Compared to the original version, the German language version does not contain the fourth item: “Awakening you during the night” because this item is not applicable in a nursing home setting since the nursing home staff is regularly awake during the whole night. The final German version was approved by R. Tractenberg, the author of the original instrument [32].

#### Secondary outcomes

##### People with dementia

The German version of the Pittsburgh Sleep Quality Index (PSQI) will be used to measure sleep quality and potential sleep problems through self-assessment of people with dementia [43]. Using 19 items, a total score of between 0 and 21 points can be determined. A value of ≥5 or higher indicates poor sleep quality. Even if it must be assumed that not all study participants are capable of self-rating (e.g. due to dementia severity), we will try to obtain the self-rating of as many people with dementia as possible. The PSQI data will be collected retrospectively during the last 4 weeks before study begin.

In addition, sleep problems will also be recorded by actigraphy on randomly selected study participants at the Lübeck study center (secondary endpoint). This will be done using non-invasive sensor technology on the wrist, enabling the recording of activity or sleep patterns [53]. Actigraphy data will be collected in the eight nursing homes participating at the Lübeck study center from 40 randomly selected people with dementia (*N* = 20 intervention group, N = 20 control group, max. of five people with dementia per nursing home). The recording of the actigraphy data will be conducted over a period of at least 3 days for each participant. The participants will wear the actigraphs from about 6 p.m. to about 10 a.m. the following day. If the data quality is insufficient on one of the days, the data collection can be extended for individual participants. In addition, information on the wearing time per recording day of each participant will be monitored in a protocol by the nurses in order to gain a better quality for the retrospective data analysis. The data will be recorded using an algorithm for a “real world scenario” [43]. Less than six hours of total nocturnal sleep time during the night will be defined as a sleep problem [32].

The Essener Questionnaire of Age and Sleepiness in the Elderly (EFAS) [54] will be used to determine retrospectively for the last 7 days whether the normal level of daytime sleepiness is exceeded (secondary endpoint). For this purpose, the four EFAS items will be proxy-rated regarding their frequency and severity (frequency and severity are multiplied). The summation of these item scores results in a total score between 0 and 48. Higher values correspond to a higher daily sleepiness [44].

Quality of life of people with dementia (secondary endpoint) will be assessed using the German version of the QUALIDEM 2.0 [45, 46]. The QUALIDEM 2.0 consists of two successive versions for mild to severe (37 items) and very severe dementia (18 items). Based on the instrument items, subscale values will be determined for the dimensions “Care relationship”, “Positive affect”, “Negative affect”, “Restless, tense behavior”, “Social relationships”, “Social isolation”, “Positive self-image”, “Feeling at home” and “Something to do” retrospectively for the last weeks [46, 55]. The latter three subscales will be omitted for people with very severe dementia. Higher subscale values correspond to a higher quality of life.

Agitated behavior of people with dementia will be recorded retrospectively for the last 2 weeks, using the German version of the Cohen Mansfield Agitation Inventory (CMAI) [56, 57]. The CMAI consists of 29 items, each rated on a 7-point scale of frequency of occurrence of agitated behaviors (proxy-based) and a 5-point scale of how disturbing the behavior is. These are recorded in terms of their frequency, their caused burden for nurses and for the people with dementia themselves. The total score ranges from 29 to 203 for frequency and 29 to 145 for burden for nurses. Higher values correspond to an increasing frequency of burden caused by agitated behavior [56, 57].

Sociodemographic data such as age, sex and care dependency level as well as data regarding accidental falls, the use of physical restraints and psychotropic medication (N05C, N05A, N05B, N06A) will be recorded from the available care documentation or based on self-developed forms with proven validity in previous studies [48, 58].

##### Nurses

The burden on nurses caused by the challenging behavior of people with dementia will be measured by the Residents’ Challenging Behavior-related Distress Index, containing nine items which are to be self-assessed by the nurses to rate their exposure to nine different types of challenging behavior of people with dementia [17]. The items are rated using a three-level response scale from “does not affect me at all” to “affects me very strongly”. The assessments per item are combined into an index from 0 to 100. Higher values are consistent with a higher burden [17].

In addition, sociodemographic variables (e.g. age, work experiences) of the nursing staff at the participating nursing homes will be assessed with single items.

##### Cost variables

Intervention costs, including costs for education and work-shop sessions (salary for nurses, material, and costs for lecturers) will be recorded on a self-developed cost sheet [48]. Costs that would not occur in routine practice (i.e., study-specific costs) will not be considered.

### Process evaluation

When evaluating complex interventions, a process evaluation is recommended for a better understanding of the implementation of the intervention [26, 29].

This process evaluation is based on the model of Grant et al. [30], which explicitly refers to cluster-randomized studies and, in addition to the various elements of process evaluation, addresses both the individual and the cluster level. Data will be collected on the following elements of process evaluation:

- ▪ Intervention components that are implemented by the research team in the nursing homes (*delivery*),
- ▪ Change processes that are implemented in nursing homes as a result of the intervention and on the basis of the developed theory of change (*response*).
- ▪ Characteristics of nursing homes and aspects of organizational culture (*context*).

Based on these three elements, we aim to collect information about the recruitment process and the reasons for participation, non-participation and withdrawal. We will also collect information about important structural and process characteristics of the clusters and the organizational culture. We will investigate the delivery of the intervention, the implementation fidelity of the intervention components as well as barriers and facilitators of the implementation, and the experiences of nurses, people with dementia and their relatives.

Data for the process evaluation will be collected continuously alongside the cRCT to investigate the process evaluation domains of *delivery*, *response* and *context*. Table 3 shows the different data sources which will be used to evaluate the recruitment and implementation process (process evaluation).

### Participant timeline

After giving informed consent, the participants’ contact data (name, nursing home unit) will be forwarded to the respective study center via a secure communication platform or phone. Baseline data will then be collected and subsequently clusters will be allocated at random to either the intervention or the control group. Recruitment will be completed prior to baseline data collection. After randomization within 14 days after baseline data collection, the assessment of the established sleep-promoting intervention will be carried out in the intervention clusters, followed by the additional intervention components (Fig. 2). After 16 weeks, the intervention will end with the second follow-up (T2) (Fig. 1).

### Sample size calculation

At follow-up (T2) we expect a difference in the prevalence of at least two sleep problems in people with dementia assessed with the SDI [32]. This difference is expected to be between 80% in the control group and 61% in the intervention group (absolute risk difference 19%). Based on Donner & Klar [59], the sample size estimation was performed for the cluster-adjusted χ^2^-test. Assuming an intra-cluster correlation coefficient (ICCC) of 0.05, a significance level of 5% and a power of 85%, an average cluster size of 15 people with dementia and sleep problems is needed; taking a possible drop-out rate of 10% during 16 weeks follow-up into account, the power will reduce to 84%.

We assume that we will not lose any clusters in the short intervention time (16 weeks).

Based on these assumptions we need 12 clusters (nursing homes) and 180 people with dementia for each group. In the case of drop outs, no new participants will be recruited.

### Assignment of interventions: allocation

#### Sequence generation

Randomization will be carried out on the cluster level. The randomization list will be computer-generated by the independent external biostatistician, who will be blinded to the identity of the participating organizations and people with dementia. The nursing homes will be allocated to the intervention or control groups using a balanced randomization using blocks, and will be stratified by the three regions Halle (Saale), Lübeck and Witten as well as the time point of the recruiting phase.

#### Concealment mechanism

This randomization list will contain pseudonymous identification numbers for the nursing homes and their allocation to group A or B. After completion of the baseline measurement of the corresponding recruiting phase, one independent researcher (not further involved in the MoNoPol-Sleep trial) from the coordinating study center in Lübeck, will electronically assign the randomization list and nursing home data for concealed allocation [60].

#### Implementation

The independent researcher from the coordinating study center in Lübeck will inform the nursing homes in written form about the group assignment. After confirmation of the group allocation by the nursing homes, the assigning person will inform the study centers about the allocation results.

### Assignment of interventions: blinding

#### Who will be blinded

Due to the type of intervention, it is not possible to blind the nursing home staff and members of the research team who are carrying out the intervention and the data collection. However, the member of the research team entering the data in the database and also the biostatistician doing the data analysis will be blinded with regard to group allocation (neutral group IDs) of clusters and people with dementia.

#### Procedure for unblinding if neede

Unblinding of the biostatistician will not be necessary.

### Data collection and management

#### Plans for assessment and collection of outcomes

The outcome instruments were chosen based on their appropriateness and feasibility for the study aim, setting and population as well as their psychometric properties (validity and reliability). The majority of the instruments are proxy-based. Nurses who have a high level of knowledge about the participating people with dementia and their care during day and night (24 h) will carry out the proxy assessment. Proxy-rating nurses have to work regularly in day and night shifts and/or take part in the handover between day and night shifts. In addition, the extent of their regular weekly working time must be at least 50%.

#### Plans to promote participant retention and complete follow-up

For adherence to the intervention see 11c. The short intervention period and the use of mainly proxy-rated measures reduces the potential burden of study participation on the people with dementia.

To support a standardized data collection, research staff, who will be trained in applying the measures prior to the data collection, will supervise the application at the proxies. The supervision will include a short instruction about the assessed construct (e.g. sleep quality, quality of life), the underlying time frame (e.g. preceding one (QUALIDEM) or 2 weeks (SDI)) and the perspective. Research staff will also support the self-assessment by the people with dementia in an interview situation and will guide the self-assessment (e.g. reading out the questions).

#### Data management

From earlier studies, various proven SOPs are available on the recruitment of nursing homes, CRF preparation according to Good Clinical Practice (GCP), data collection, data entry, data audit, ethical issue management, as well as on informed consent by care dependent persons and legal guardians [48, 58].

The transfer of the data will be between secure servers (encrypted data transfer). Any hard-copy printouts, USB data versions or other removable media being used to transfer protected health information will be destroyed after transmission is complete.

A careful plausibility check will be carried out before the data is transferred to the biostatistician. The biostatistician will carry out additional logical plausibility checks.

### Confidentiality

The data from the nursing homes and the people with dementia will be recorded electronically and pseudonymized. Code lists and data will be stored in each study center on different, separate and secure servers. Only persons involved in the study will have access to these secure servers. All of the data will be checked for inconsistency and completeness.

After completion of the measurement point T2, the data will be anonymized at the respective study center. The code lists with the assignment of the identity data to the pseudonym will subsequently be deleted.

### Plans for collection, laboratory evaluation and storage of biological specimens for genetic or molecular analysis in this trial/future use

Not applicable, as no biological specimens will be collected as part of this trial.

### Statistical methods

#### Statistical methods for primary and secondary outcomes

The statistical analysis will follow GCP [61] standards and will be conducted by the blinded biostatistician (BH) after the end of follow-up (T2). Data analysis will be performed according to the intention-to-treat (ITT) principle. For the primary outcome, the prevalence of ≥2 sleep problems (measured using the SDI) will be compared between the intervention and control groups in the population by applying a two sided cluster-adjusted χ^2^-test at a level of significance of α = 0.05 [59]. The corresponding cluster-adjusted 95% confidence intervals will be calculated.

Secondary endpoints will be compared between the intervention and control groups by using cluster-adjusted tests depending on their distribution. Additional analyses of longitudinal data, which include repeated measures per participant or potential risk factors together with initial values at T0, are planned and will use mixed (generalized) linear models (covariance pattern: unstructured by group; type of model depending on the distribution of the outcome). Secondary outcomes of the nurses will be analyzed and clusters adjusted using similar methods. Costs between study groups will be compared descriptively using averages, standard deviation and quartiles without employing any further cost evaluation approach.

#### Interim analyses

No interim analyses are planned.

#### Methods for additional analyses (e.g. subgroup analysis)

No additional analyses are planned.

#### Methods in analysis to handle protocol non-adherence and any statistical methods to handle missing data

The primary analysis follows the intention-to-treat principle. In the secondary analyses by mixed models using all the values in the time course, an adjustment for missing values - especially through dropouts - will be made. Multiple imputations will not be performed. The primary analysis will be performed as a secondary per protocol analysis after excluding participants following protocol violations.

#### Process evaluation

The analysis of process data will be blinded to the results of the effect data. Quantitative data will be analyzed descriptively. Qualitative data (interview and focus group data as well as document analyses) will be analyzed based on the principles of qualitative content analysis [62]. Interview data will be digitally recorded and transcribed verbatim. 10% of the process documents and qualitative interviews will be analyzed independently by two researchers. Based on these analyses, all further analyses will be performed by one researcher. A peer group will be available to help the respective researcher reflect any uncertainties. In addition, the interim results of the qualitative analyses will be regularly presented to the research team and discussed critically. The results of the process evaluation will be summarized and described narratively.

### Oversight and monitoring

#### Composition of the coordinating center and trial steering committee

The steering committee will be composed of experts and important stakeholders in the field of research for people with dementia in nursing homes. The committee has met every two to 4 months during the development of the MoNoPol-Sleep intervention and the planning of the MoNoPol-Sleep trial. During the trial, the committee will meet via weekly or monthly telephone conferences in order to review the progress of the trial and to suggest decisions within the framework of the study. All steering committee members must agree to the final protocol before publication. The following persons represent the committee:

- ▪ Prof. Dr. Sascha Köpke, Institute of Nursing Science, University of Cologne, Germany (Scientific coordinator)
- ▪ Dr. Martin N. Dichter, Institute of Nursing Science, University of Cologne, Germany
- ▪ Prof. Dr. Gabriele Meyer, Institute for Health and Nursing Science, Martin Luther University Halle-Wittenberg, Halle (Saale), Germany
- ▪ Dr. Almuth Berg, Institute for Health and Nursing Science, Martin Luther University Halle-Wittenberg, Halle (Saale), Germany
- ▪ Prof. Dr. Margareta Halek, German Center for Neurodegenerative Diseases (DZNE), Witten, Germany

#### Composition of the data monitoring committee, its role and reporting structure

The coordinating study center (University of Lübeck) will supervise data collection, data management and data quality issues. The scientific coordinator will also submit bi-monthly reports to the scientific advisory board (see below) for external audit, who will verify the reports for adherence to the study protocol and standards of GCP. Data monitoring will increase the credibility of the study and help to improve the data collection and archiving procedures. It will be carried out according to a data monitoring manual which follows GCP rules and has been developed and proven in eight countries during a current European study [63]. Due to our own substantial experience, the data audit will not be conducted by a company or an external organization in order to avoid the study becoming unreasonably expensive. The data audit will be carried out by extensively trained researchers who are not engaged in the study.

The study will be supervised by an external advisory board with different fields of expertise and perspectives:

- ▪ Ms. Brigitte Bührlen, Munich, patient advocate, representative of “Wir pflegen! Stiftung pflegender Angehöriger” (self-help organization for people with dementia and/or their informal caregivers), Munich, Germany
- ▪ Prof. Dr. med. Helmut Frohnhofen, geriatrician and sleep physician, University Hospital Düsseldorf, Düsseldorf, Germany
- ▪ Mr. Thomas Peters, nursing home manager, Arbeiter Samariter Bund, “Norbert Burger nursing home”, Cologne, Germany
- ▪ Dr. med. Tanja Richter, physician, legal guardian, health services researcher, University of Hamburg, Faculty of Mathematics, Informatics and Natural Sciences, Hamburg, Germany

#### Adverse event reporting and harms

Given the nature of the intervention, we do not expect serious adverse events. Thus, no interim analysis is planned and no termination rules will be applied.

#### Frequency and plans for auditing trial conduct

The study will be planned, implemented and evaluated in accordance with the principles of ICH-GCP and the Declaration of Helsinki [64]. Concordant study procedures will be performed in all three study centers.

#### Plans for communicating important protocol amendments to relevant parties (e.g. trial participants, ethical committees)

The ethics committee will be informed immediately about any protocol amendments and serious or unexpected adverse events as well as a premature end of the study. All changes will be noted in the study registration.

### Dissemination plans

The main study results will be published in an international, peer reviewed journal and will be presented at relevant scientific conferences. All the results will be reported based on this study protocol as well as the recommendations of the CONSORT statement extended to cluster-randomized trials [65] and the CReDECI 2 guideline [66].

Authorship will be shared between persons involved in the study following the current guidelines of the International Committee of Medical Journal Editors (ICMJE). Persons not directly involved in the study will not be granted authorship.

## Discussion

This cluster-randomized controlled trial will investigate the effectiveness of a complex intervention to avoid and reduce sleep problems in people with dementia living in nursing homes. We do not assume negative effects for the study participants. We expect a significant reduction of sleep problems of the participating people with dementia when compared to the control group. We also expect that the process evaluation results of this study will enrich our knowledge of promoting factors and obstacles to the implementation of this complex intervention. If implemented successfully, the updated program will comprise a less extensive and potentially less costly intervention to avoid and reduce sleep problems in people with dementia. The comprehensive assessment of process measures and the cost analysis can increase the knowledge base for the further development of the intervention. During the study, the German version of the PSQI as well as the SDI will be investigated regarding their psychometric properties (reliability and validity), which will provide important insights into the use of these instruments for the German versions.

## Data Availability

Not applicable.

## Abbreviations

AITCS: Assessment of Interprofessional Team Collaboration Scale
CMAI: Cohen-Mansfield Agitation Inventory
cRct: Cluster-randomized controlled trial
CRF: Case report form
DSS: Dementia Screening Scale
EFAS: Essener Questionnaire of Age and Sleepiness in the Elderly
GCP: Good clinical practice
ICCC: Intra-cluster correlation coefficient
ICH-GCP: International Conference of Harmonization – good clinical practice
MoNoPol-Sleep: Multi-modal, non-pharmacological intervention for sleep disturbances in people with dementia living in nursing homes
MRC: UK Medical Research Council
NPI-NH: Neuropsychiatric Inventory - Nursing Home
ORIC: Organizational Readiness for Implementing Changes
PCQ-S: Person-centered climate questionnaire-staff version
PSQI: Pittsburgh Sleep Quality Index
REM: Rapid-Eye-Movement
SDI: Sleep Disorder Inventory
SOP: Standard Operation Procedure
